# An Ensemble Deep Learning Approach for EEG-Based Emotion Recognition Using Multi-Class CSP

**DOI:** 10.3390/biomimetics9120761

**Published:** 2024-12-14

**Authors:** Behzad Yousefipour, Vahid Rajabpour, Hamidreza Abdoljabbari, Sobhan Sheykhivand, Sebelan Danishvar

**Affiliations:** 1 Department of Electrical Engineering, Sharif University of Technology, Tehran 51666-16471, Iran; 2 Faculty of Electrical and Computer Engineering, University of Tabriz, Tabriz 51666-16471, Iran; 3 School of Electrical Engineering, Iran University of Science and Technology (IUST), Tehran 51666-16471, Iran; 4 Department of Biomedical Engineering, University of Bonab, Bonab 55517-61167, Iran; 5 College of Engineering, Design and Physical Sciences, Brunel University London, Uxbridge UB8 3PH, UK

**Keywords:** Auto Encoder (AE), Brain–Computer Interface (BCI), Convolutional Neural Network (CNN), Electroencephalogram (EEG), emotion detection, ensemble deep learning, multi-class common spatial pattern (MCCSP)

## Abstract

In recent years, significant advancements have been made in the field of brain–computer interfaces (BCIs), particularly in the area of emotion recognition using EEG signals. The majority of earlier research in this field has missed the spatial–temporal characteristics of EEG signals, which are critical for accurate emotion recognition. In this study, a novel approach is presented for classifying emotions into three categories, positive, negative, and neutral, using a custom-collected dataset. The dataset used in this study was specifically collected for this purpose from 16 participants, comprising EEG recordings corresponding to the three emotional states induced by musical stimuli. A multi-class Common Spatial Pattern (MCCSP) technique was employed for the processing stage of the EEG signals. These processed signals were then fed into an ensemble model comprising three autoencoders with Convolutional Neural Network (CNN) layers. A classification accuracy of 99.44 ± 0.39% for the three emotional classes was achieved by the proposed method. This performance surpasses previous studies, demonstrating the effectiveness of the approach. The high accuracy indicates that the method could be a promising candidate for future BCI applications, providing a reliable means of emotion detection.

## 1. Introduction

The complex interactions between physiological and cognitive processes, known as emotions, play a crucial role in shaping human behavior and experiences [1]. These interactions have driven significant progress in biomedical engineering, artificial intelligence, and neuroscience [2,3]. In artificial intelligence [4,5], understanding and modeling these interactions have led to advancements in emotion-recognition systems, enabling machines to better interpret and respond to human emotional states. Based on two primary scientific viewpoints, emotions are examined using cognitive appraisal theory, which views them as assessments of how well current conditions match with personal goals or well-being [6]. Alternatively, the James–Lange theory suggests that emotions arise from the perception of physiological changes, such as variations in heart rate, respiration, tears, and hormone composition [7]. This dual perspective highlights the conscious and intensely mental nature of emotions, characterized by varying degrees of pleasure or displeasure.

**Figure 1:**
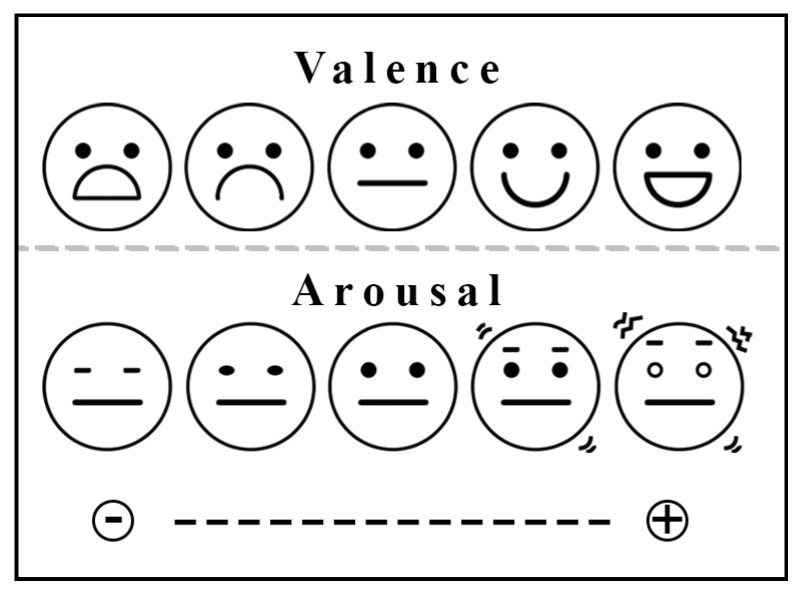
Set of emotions based on the valence and arousal dimensions.

In general terms, emotions can be categorized into two main perspectives: discrete and dimensional. The discrete perspective asserts that humans possess a core set of basic emotions, as exemplified by Ekman’s six: anger, disgust, fear, happiness, sadness, and surprise [8,9]. Meanwhile, the dimensional approach categorizes emotions along the dimensions of valence, arousal, and dominance [10]. Valence describes the intrinsic attractiveness or unpleasantness of a situation or stimuli, which ranges from negative to positive and reflects an individual’s level of aversion or favorability. Arousal, on the other hand, refers to the level of physiological and psychological activation elicited by an emotional state, ranging from lethargy (low activation) to excitement (high activation) [11]. Dominance indicates the perceived level of control or power associated with an emotion. This model allows for a detailed characterization of emotions beyond discrete categories, enabling a deeper understanding of human affective experiences. Figure 1 illustrates a range (set) of emoticons that represent various emotional states, which are used to convey the dimensional approach to emotions.

Emotion research employs a variety of methodologies, including cognitive assessments, physiological measurements, and subjective self-reports [12]. Researchers use diverse stimuli, such as music [13], images, and movies [14], to elicit emotions in controlled conditions. Music, in particular, is noted for its efficacy in inducing emotional responses. Tools like the Self-Assessment Manikin (SAM) are often used to measure subjective experiences of valence, arousal, and dominance, enhancing our understanding of emotional nuances [15]. Consequently, the study of emotions necessitates an interdisciplinary approach that encompasses physiological, cognitive, and behavioral aspects.

Emotion recognition is crucial for human–computer interaction (HCI) systems, enabling computers to better understand and respond to users’ affective states [16]. Despite the rapid integration of computers into our daily life, they still lack the ability to comprehend human emotions, which hinders the effectiveness of HCI. Identifying a user’s emotional state facilitates more natural and personalized interactions, applicable in education, entertainment, and healthcare [17,18].

Existing emotion-recognition research concentrates on two primary categories: (1) non-physiological signals like facial expressions, speech, and gestures [19,20,21,22], and (2) physiological signals such as Electrocardiogram (ECG), Electromyogram (EMG), Galvanic Skin Response (GSR), and Respiration (RS) [23,24,25,26,27]. While non-physiological methods are cost-effective and easy to implement, their reliability can be compromised by individuals concealing their emotions. Physiological signals, particularly EEG, offer more reliable insights into underlying emotional responses due to their ability to capture real-time neuronal activity [28,29].

EEG is a widely used technique in brain mapping and neuroimaging, which quantifies the electrical fields generated by brain activity with high temporal resolution [30]. By capturing electric potential differences on the scalp, EEG reflects neuronal activity and has been instrumental in various clinical applications such as studying sleep patterns [31] and epilepsy [32]. EEG is also valuable in neuroscience and psychology research, providing opportunities for studying cognitive functions, affective monitoring, and brain–computer interfaces (BCIs). Several datasets exist for emotion recognition using EEG signals. Notable examples include DEAP (which combines EEG, physiological, and video signals) [33], DREAMER (focused on EEG and ECG signals from affordable off-the-shelf devices) [34], SEED (with three emotion classes) [35], and SEED-IV (which expands SEED to four emotion classes) [35].

EEG signals are susceptible to noise from external sources and other physiological activities, resulting in a low signal-to-noise ratio [36]. Furthermore, EEG data exhibit nonlinear and non-stationary characteristics [37], posing challenges for traditional emotion-recognition models.

To address these challenges and enhance the performance and generalizability of EEG-based emotion-recognition models, novel approaches are essential. Ensemble learning offers a promising solution [38,39]. In recent years, the application of ensemble learning methods for emotion recognition has garnered significant attention and demonstrated remarkable success [40,41,42].

This study investigates the complex interplay between music, emotions, and the brain. Music, chosen for its universal ability to induce emotions, was used to explore its general and physiological effects on individuals with varying mental and emotional backgrounds. To account for individual differences, we selected music based on the participants’ cultural context (Iranian), using historical themes for positive emotions and sad themes for negative emotions. This approach acknowledges that the impact of music depends on the listener’s neuronal condition, mental history, and listening habits. Our meticulously designed experimental framework integrates psychometric assessments (BDI and SAM tests), EEG recordings, and carefully chosen music stimuli. Combining these data sources will allow us to elucidate the neural substrates of emotional processing triggered by music, ultimately advancing our understanding of affective neuroscience and potentially opening the way for advancements in music therapy.

## 2. Literature Review

In [43], the authors extracted features from preprocessed EEG data, considering time domain, time-frequency domain, and nonlinear features related to emotion. They used Linear discriminant analysis (LDA) for feature selection and trained a classifier using the ensemble learning method, AdaBoost, for binary classification. Their approach achieved an average accuracy rate of up to 88.70% on the dominance dimension in the DEAP dataset. A new ensemble learning method with multiple objective particle swarm optimization was introduced in [44]. Key steps included feature extraction from preprocessed EEG data using a 4 s sliding time window with a 2 s overlap, resulting in a feature vector. L1 regularization was applied for effective feature selection, followed by model selection to identify optimal sub-models. An ensemble operator converted single model classification results from discrete to continuous values. The ensemble parameters were optimized using multiple objective particle swarm optimization, and the approach was evaluated on DEAP and SEED datasets, achieving improved recognition performance with average accuracies of 65.70% for arousal and 64.22% for valence (DEAP) and the average accuracy on the SEED database is 84.44%. In [45], ensemble learning-based machine learning (EML) algorithms were compared with conventional machine learning (CML) algorithms for emotion recognition using the DREAMER database. They separated EEG signals into theta, alpha, beta, and gamma bands using discrete wavelet transform (DWT), followed by empirical mode decomposition. Thirty-one statistical features were extracted from the intrinsic mode functions (IMFs). EML algorithms (including bagging, random forest, rotation random forest, extreme gradient boost, and adaptive boosting) outperformed CML algorithms in terms of mean accuracy for both arousal (88.95% vs. 83.08%) and valence (88.90% vs. 82.81%) dimensions. In [46], a method called Multi-Scale Frequency Bands Ensemble Learning (MSFBEL) was proposed. EEG frequency bands were reorganized into several local scales and one global scale, with a base classifier trained on each scale. They designed an adaptive weight learning method to assign larger weights to more important scales, effectively fusing complementary information. This approach achieved average accuracies of 82.75%, 87.87%, and 78.27% across three sessions on the SEED-IV dataset, and an average accuracy of 74.22% in four-category classification under 5-fold cross-validation on the DEAP dataset. In [47], three nonlinear features and eight ensemble learning approaches were proposed to predict six basic emotions. They utilized a randomized grid search technique for tuning the hyperparameters of each algorithm to increase the recognition rate. The synthetic minority oversampling technique (SMOTE) was used to handle the imbalanced sample distribution of each emotion. Their highest average accuracy was achieved at 89.38% using Higuchi fractal dimension on the DEAP dataset. In [48], a multi-scale principal component analysis and symlets-4 filter were used for the preprocessing stage. They utilized a version of DWT, namely dual-tree complex wavelet transform (DTCWT), for the feature extraction. Various statistical criteria were applied to reduce feature dimension size. This framework achieved nearly 96.8% accuracy using a random subspace ensemble classifier on the SEED dataset. In [49], a method was developed where they derived combinations of all adjacent frequency bands at various scales through a process of permutation and reorganization. They employed a classification approach known as homogeneous-collaboration-representation to obtain the classification outcomes for each combination. In the final step, they introduced a circular multi-grained ensemble learning method to re-extract the features of each result and combined the machine learning methods with a simple majority voting system for decision fusion. Their proposed framework achieved an accuracy of 95.09% and 94.38% in arousal and valence, respectively, on the DEAP dataset, and 96.37% accuracy on the SEED-IV dataset.

Deep learning methods offer significant advantages over traditional machine learning techniques, especially in emotion detection and classification from EEG signals [50]. They automatically extract complex features from raw data, eliminating the need for time-consuming manual feature engineering. In EEG signal processing, deep learning models like CNNs and Long Short-Term Memory networks (LSTMs) excel in capturing spatial and temporal patterns, demonstrating superior accuracy and robustness [51]. This high performance is due to their ability to learn from large datasets, effectively capturing intricate patterns essential for accurate emotion classification. While traditional methods have their merits, deep learning is particularly adept at managing the complex and high-dimensional nature of EEG signals, often leading to superior performance and reduced manual intervention. Moreover, deep learning algorithms can integrate feature extraction, data preprocessing, and classification within a single framework, simplifying processing pipelines and enhancing overall performance. The success of deep learning in managing complex data across various domains, such as images, text, and audio signals, motivates its application to EEG-based emotion recognition [52,53]. Deep learning is currently being used in hot topics such as COVID-19 [54,55], speculative hype [56], password meter [57], social media [58], music [59], tackling domain shifts [60], modeling analysis [61], image and attention detection [62,63], postoperative intensive care unit [64], predictive [65,66], mathematical modeling [67], risk behavior [68,69], peer assessment [70], optimizing [71], multi objective [72,73], human decision [74].

In [75], the authors utilized a 3D-CNN architecture to extract spatial–temporal features from EEG and facial data in the DEAP dataset. Data augmentation and ensemble learning techniques improved fusion predictions, resulting in recognition accuracies of 96.13% for valence and 96.79% for arousal classes. In [76], the authors proposed an approach that leveraged dynamic entropy measurements derived from EEG signals, capturing consecutive entropy values over time to enhance the characterization of emotional patterns. By combining ensemble learning techniques with a recurrent neural network (RNN), their approach achieved the highest average accuracy of 94.67% for distinguishing negative and positive emotions in the DEAP dataset. In [77], the authors computed differential entropy over five frequency bands extracted from EEG signals and developed a hybrid model based on CNN and LSTM. The extracted features were fed to all three models (CNN, LSTM, and hybrid). An ensemble model then combined the predictions of all three models. Their proposed approach was validated on two datasets (SEED and DEAP), and achieved 97.16% accuracy on the SEED dataset for emotion classification. The authors in [75], used the Continuous Wavelet Transform (CWT) [78] approach to generate scalograms from one-dimensional EEG signals. Then, five different CNNs—AlexNet, VGG-19, Inception-v1, ResNet-18, and Inception-v3—were retrained using these images. The classification of emotional state was determined using a majority voting procedure. Their approach attained an accuracy of 96.90% in identifying four emotional states in the DEAP dataset.

Numerous studies have investigated automatic emotion recognition based on EEG signals, each of them bringing valuable insights to the field. However, many of these studies have limitations. Earlier studies overlooked spatial information between electrodes, despite its importance as an input factor. Furthermore, while there are existing databases primarily based on visual stimulation, they are not necessarily optimal for all deep learning networks. To address the specific needs and goals of our research, we created a dataset focused exclusively on auditory stimulation, which was carefully designed to align with the specific objectives of our study. This decision demonstrates our determination to personalize the dataset to our study concerns rather than depending simply on existing public databases, which may not have entirely satisfied our needs.

Consequently, this research aims to address these limitations by introducing a novel model that balances high reliability and low computational complexity for automatic emotion recognition. To achieve this, a new database focusing on emotion detection utilizing musical stimuli was carefully collected at Tabriz University’s BCI laboratory, while following all necessary standards. The proposed model combines multi-class common spatial patterns (MCCSPs) with ensemble deep learning, effectively identifying optimal features from recorded EEG signals to classify basic emotions into three distinct classes (positive, neutral, and negative). The study’s contributions are structured as follows:

- A new database for emotion recognition using musical stimuli based on EEG signals was collected.
- The combination of MCCSPs and ensemble deep learning, incorporating autoencoders with 2D-CNN layers, was used to extract features from spatio–temporal 2D representations of EEG signals. This approach led to the elimination of the feature selection block diagram.
- An algorithm based on ensemble deep learning was provided, designed to be resilient to environmental noise.
- In addition to the traditional fully connected layers (used for classification after feature extraction from the ensemble model), other major classifiers—k-nearest neighbors (KNNs), support vector machines (SVMs), and multilayer perceptrons (MLPs)—were also employed.
- An automatic model was presented, achieving superior accuracy and minimal error in classifying three emotional classes compared to prior research.

In the subsequent sections, we delve into the details of our research. Section 3 covers materials and methods, including data collection, outlining data preprocessing, the mathematical foundations relevant to MCCSPs, and deep learning networks. We then present simulation results and compare them with prior research in Section 4. Section 5 explores applications related to our current study, and finally, Section 6 provides the conclusion.

## 3. Materials and Methods

**Figure 2:**
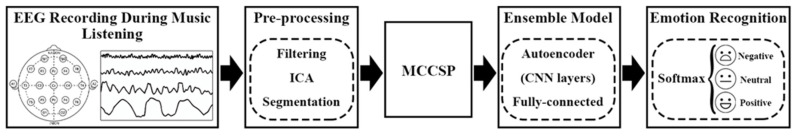
The main framework of the proposed model.

In this section, we begin by detailing the data-collection process. Subsequently, we cover data preparation and preprocessing, explore the mathematical foundations of the MCCSPs approach, discuss the design of the ensemble deep network architecture, address hyperparameters and network training details, and describe the training and evaluation sets. The main structure of our proposed model for automatic emotion identification from EEG signals using musical stimulation is depicted in Figure 2.

### 3.1. Data Collecting

Our work sought to build an EEG-based emotion-recognition database that includes positive, negative, and neutral emotions. In order to achieve robust emotion classification, we employed a multi-modal approach combining physiological data (EEG) with self-reported emotional experiences using the SAM questionnaire test. The University of Tabriz’s ethics committee approved the study procedure, which was carried out in the university’s BCI lab (Faculty of Electrical and Computer Engineering), in accordance with all ethical standards. Participants provided written informed consent and underwent a health screening prior to data collection. The consent form ensured no history of mental illness or epilepsy, no use of psychiatric drugs, normal sleep patterns, and avoidance of fatty foods, caffeine, and hair washing before the test. Participants also completed the Beck Depression Inventory (BDI); those scoring above 21 were excluded to prevent the inhibition of emotional induction, as per psychological standards. To ensure the reliability of self-reported emotional experiences, participants completed the nine-point SAM test before and after each music track, reporting their emotional valence (positive/negative) and arousal (high/low) on a defined scale. Scores below 3 on the SAM test were considered low, and scores above 6 were considered high.

Sixteen individuals (6 females and 10 males) aged 20 to 28 participated in the experiment. Emotions were induced through music stimulation, with tracks selected for their emotional content: sad music for negative emotion induction and traditional/historical music for positive emotions. Each track was played for one minute, followed by a 15 s pause to prevent emotional carryover. Additionally, a neutral state was incorporated into the process. Participants listened via headphones to enhance the induction process.

EEG signals were recorded using a 21-channel Medicom device, standardized according to the 10–20 system (Fp1, Fp2, F7, F3, Fz, F4, F8, A1, T3, C3, Cz, C4, T4, A2, T5, P3, Pz, P4, T6, O1, O2). Silver-chloride electrodes were arranged in a cap configuration to facilitate data acquisition. All channel data were referenced to the A1 and A2 electrodes, digitized at 250 Hz, with an impedance matching of 10 kΩ on the electrodes. We used a bipolar recording mode to improve the quality of the signal.

**Figure 3:**
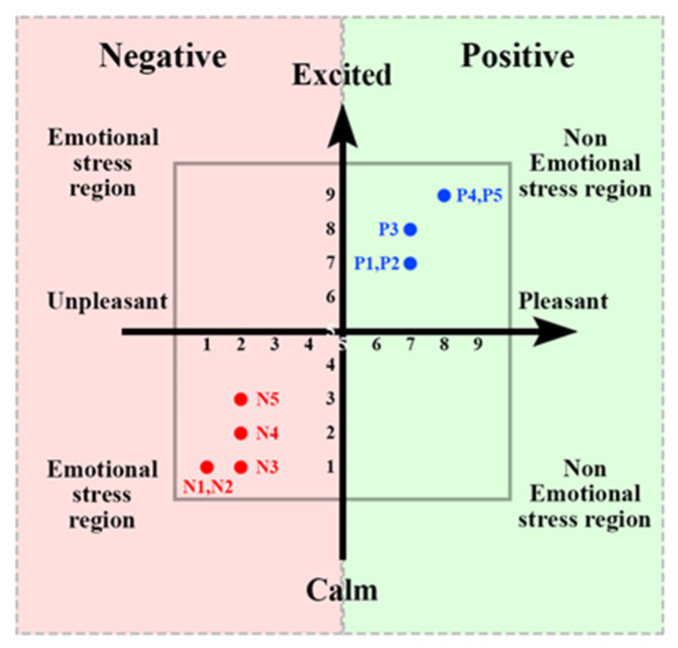
Validation of SAM test.

**Figure 4:**
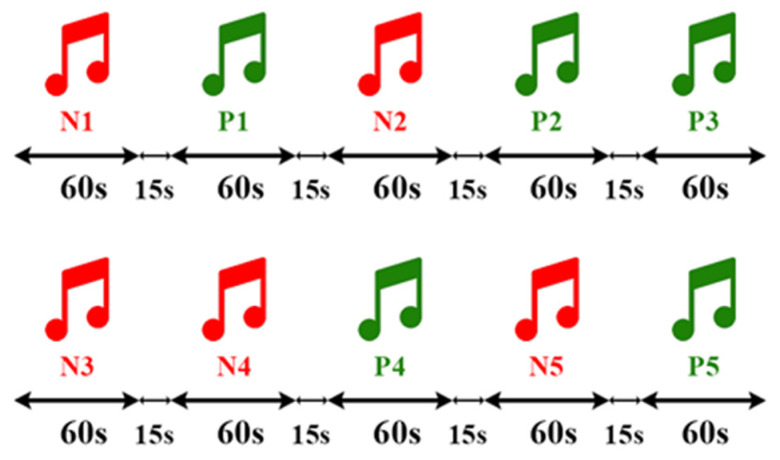
The duration and order of the music tracks.

**Figure 5:**
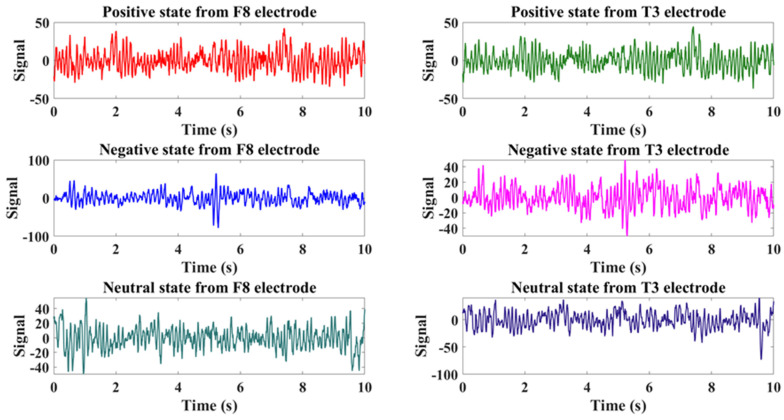
Part of the EEG signal for positive, negative, and neutral stages of T3 and F8 channels for Subject 4.

Table 1 summarizes the descriptive statistics for the BDI and SAM scores and justifications for exclusions of participants (e.g., Subject 3 was excluded because of mismatched SAM ratings). The results of the SAM test validation are presented in Figure 3. The entire experiment lasted approximately 12 min (720 s). The music playback order is shown in Figure 4. The Persian songs that were played for the subjects are listed in Table 2. To prevent the brain from habituating to a task over time, which can occur if the task follows a repetitive pattern, it is necessary to introduce random stimulation. Habit formation can result in signals that are not due to genuine stimulation but rather the brain’s adaptation to the task. Therefore, to avoid generating such signals, it is essential to apply stimulation in a random manner. As shown in Figure 4 and Table 2, this principle is observed by playing music with positive emotions followed by two pieces of music with negative emotions in a randomized sequence. Figure 5 also shows samples of EEG signals for the three stages of emotion for T3 and F8 channels on Subject 4.

### 3.2. Preprocessing

In the preprocessing of EEG signals, a multi-step approach was employed to ensure the integrity and quality of the data. Initially, a notch filter was applied to remove the 50 Hz frequency of the power supply, which is a common source of electrical interference in EEG recordings. Subsequently, a Finite Impulse Response (FIR) filter was utilized within the EEGlab environment, with a passband set between 0.5 and 45 Hz. This filtering range was chosen to retain the frequency components most relevant to cognitive processes while excluding high-frequency noise and slow drifts. Following the filtering, Independent Component Analysis (ICA) was conducted using the “runica” command, a standard procedure in EEGlab for isolating and removing artifacts from the EEG data. ICA is particularly effective in identifying components associated with eye movements and blinks, as well as other non-brain activities. The next step in the preprocessing pipeline involved the visual inspection of the data to manually identify and remove any remaining artifacts from muscle movements or other sources of noise. The careful procedure guaranteed that the resulting EEG data were devoid of artifacts and appropriate for further analysis.

Because the first part of each 60 s signal in the positive and negative classes may be affected by the previous event, we removed the first 10 s [44]. Also, we removed the last 10 s due to lack of specific feeling or in other words saturation of emotions in the second half of the signals. Therefore, the final signal for each of the positive and negative classes was 200 s. For the neutral class, no deletions were made, and considering that the signal was recorded eight times without playing music, we had 120 s of data for the neutral class. Figure 4 illustrates that the neutral class has fewer data points compared to the positive and negative classes, leading to a data imbalance. This imbalance can potentially cause overfitting and introduce bias into classification results, reducing overall accuracy. To address the issue of class imbalance, we employed overlapping techniques. During this procedure, epochs corresponding to each emotion were merged to create an extended continuous signal. Rectangular windows with predetermined lengths and overlaps were then applied to ensure that the number of epochs gathered for each emotion category was equalized.

In the proposed method, each channel contains 200 s of signals for both the positive and negative classes, and 120 s for the neutral class. To prevent overfitting, the overlap method segmented the data into 3 s sections with a 70% overlap for the positive and negative classes, and an 86% overlap for the neutral class, compensating for fewer data points in the neutral class. Based on the sampling rate, segmenting each channel resulted in 219 × 750 data points for both the positive and negative classes, and 208 × 750 data points for the neutral class. With 7 subjects and 19 channels involved, the final dimensions of the input tensor were (7 × 219) × 750 × 19 for the positive and negative classes, and (7 × 208) × 750 × 19 for the neutral class. This amounted to 1533 matrices of 750 × 19 for the positive and negative classes and 1456 matrices of 750 × 19 for the neutral class.

During the final step of preprocessing, the signal underwent decomposition into frequency bands delta (0.5–4 Hz), theta (4–8 Hz), alpha (8–13 Hz), beta (13–30 Hz), and gamma (30–45 Hz) using an FIR filter with an order of 800. The specific ranges for each band were selected subjectively. The entire frequency range (all-band) was 0.5 to 45 Hz.

### 3.3. MCCSP

**Figure 6:**
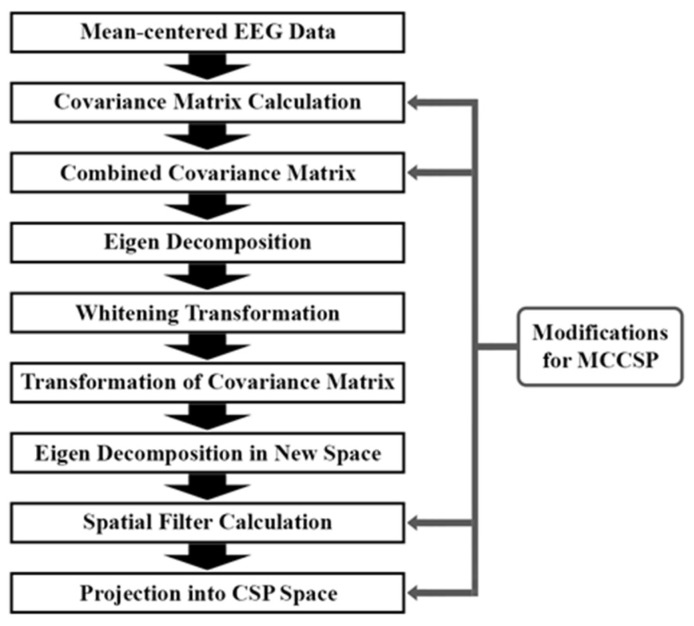
The MCCSP process flowchart.

In the proposed study, the CSP algorithm provides inputs to feature extraction methods. This study employs the MCCSP algorithm, which extends spatial patterns to data comprising multiple classes [79,80]. Unlike two-class CSPs, MCCSPs involve additional steps, including class-specific covariance matrix calculations, combining these into a multi-class covariance matrix, generalizing spatial filters across classes, and performing projections for each class to ensure multi-class discrimination. The main idea of this algorithm is to increase the disparity between classes of EEG data by using a projection matrix that converts the data into a spatial space with fewer dimensions [81]. The flowchart of the MCCSP process is shown in Figure 6.

Given our objective to categorize EEG signals based on emotions into three classes, positive, negative, and neutral, we initially subtract the mean from the raw EEG data of each class. This results in ${{[X}_{i,j}]}_{T\times C}$ for i=1, 2, 3 and j=1, 2, …, N, where i and j are the index of the class number and sample number, respectively. Here, T indicates the number of samples in each segment, C stands for the number of EEG channels, and N is the maximum number of samples according to the number of classes (for first and second classes—positive and negative—N=1533, and for third class—neutral—N=1456). This procedure, known as common average referencing, is typically carried out to eliminate noise. Subsequently, we calculate the covariance matrix for each class as shown in Equation (1):

(1) $$R_{i}=\sum_{j=1}^{N}{X_{i,j}}^{T}X_{i,j},\;\left\{\begin{array}{c} i=1,\;2,\;3\\ j=1,\;2,\;\ldots ,\;N \end{array}\right.$$

For each of the three classes, denoted by the index i, we compute a distinct covariance matrix. The notation $X^{T}$ represents the transpose of matrix X. The combined covariance matrix is then derived by summing the individual covariance matrices across all classes as shown in Equation (2):

(2) $$R=\sum_{i=1}^{3}R_{i},$$

Decomposition of the combined covariance matrix to obtain its eigenvalues and eigenvectors is outlined in Equation (3):

(3) $$R=U_{0}\Lambda {U_{0}}^{T},$$

The matrix $U_{0}$ is an C×C unitary matrix that holds the principal components, and Λ is a C×C diagonal matrix with the eigenvalues. Consequently, we construct the whitening transformation matrix as presented in Equation (4):

(4) $$W=\Lambda^{-1/2}{U_{0}}^{T},$$

This procedure identifies the components associated with non-zero eigenvalues. We then transform the covariance matrix $R_{i}$ into $S_{i}$ using the mapping defined below:

(5) $$S_{i}=WR_{i}W^{T},$$

The variable i denotes the class number. We determine the spatial values and vectors of the covariance matrix within the new space $S_{i}$ for class i in the following manner. The eigen decomposition of the covariance matrix $S_{i}$ in this new space for the i-th class is expressed as Equation (6):

(6) $$S_{i}=U_{i}\Lambda_{i}{U_{i}}^{T},\;i=1,\;2,\;3$$

The matrix $U_{i}$ is the common principal components matrix for class i. We select m principal components from $S_{i}$ that correspond to the largest eigenvalues, and similarly, m principal components associated with the smallest eigenvalues are chosen, which are represented by $U_{i}^{s}$. The spatial filter for the i-th class is then formulated as Equation (7):

(7) $$SF_{i}={\left(U_{i}^{s}\right)}^{T}W,\;i=1,\;2,\;3$$

where $SF_{i}$ denotes the spatial filter for class i. Following the acquisition of this spatial filter, we can express the decomposition of $X_{i,j}$ as Equation (8):

(8) $$X_{i}=SP_{i}Z_{i},\;i=1,\;2,\;3$$

The term $SP_{i}$ signifies the pseudoinverse of $SF_{i}$, which is interpreted as the spatial patterns matrix for the i-th class. The variable $Z_{i}$ represents a new time series created by projecting $X_{i,j}$ onto the CSP space, and this relationship is formulated as follows:

(9) $$Z_{i}=SF_{i}X_{i,j},\;j=1,\;2,\;\ldots ,\;N$$

where $X_{i}$ represents the EEG data for the i-th class, and $SF_{i}$ is the associated spatial filter obtained from Equation (7). $Z_{i}$, which is the output of the MCCSP algorithm, is used as input for the next step.

We iterate the procedure across all emotion classes to acquire spatial filters for each one. Then, utilizing these spatial patterns, we extract feature vectors for every class, which serve as the output from the MCCSP method. The core principle of MCCSP, when applied to more than two conditions, is its ability to calculate spatial patterns for an individual class in contrast to all other conditions. The MCCSP algorithm functions similarly to CSP, aiming to minimize variance across all classes except the target class, while maximizing variance within the target class. This process enables MCCSP to yield information that is more easily distinguishable between classes.

The output dimensions of the MCCSP remain unchanged and no dimension reduction has taken place. In the following phases, we used the results from the MCCSP as a foundation for our feature extraction techniques.

### 3.4. Ensemble Model

**Figure 7:**
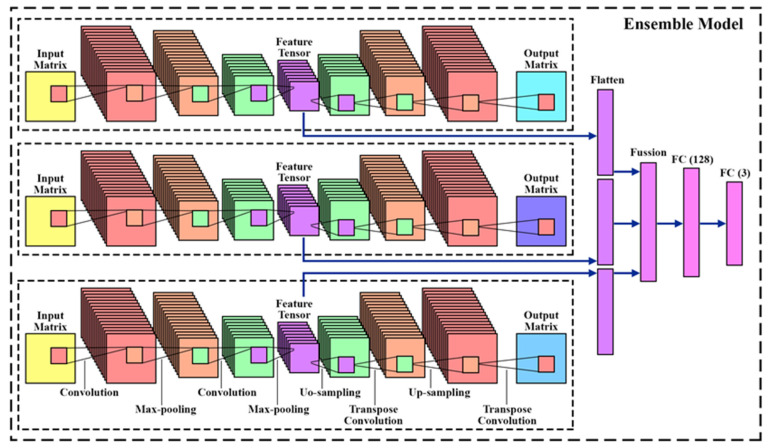
Proposed ensemble model.

Our ensemble learning framework employs a parallel structure, with identical datasets shared across three sub-networks CNN-Autoencoder (CNN-AE), as depicted in Figure 7. Each sub-network processes the data through convolutional layers with 16 filters in the first layer and 8 filters in the second layer, followed by maximum pooling. The outputs of these layers provide encoded features, which are then processed by the autoencoders. The first section of the ensemble model consists of three autoencoders, each receiving the same input data. The encoded features from these autoencoders are flattened and concatenated. This design ensures that the ensemble model effectively captures the complexity of EEG signals for emotion recognition, enhancing robustness and accuracy. The concatenated features are then input into a fully connected network with 128 neurons. This network reduces the feature dimensionality before passing the data through a SoftMax layer with 3 neurons, each representing one of the three emotional states: positive, negative, and neutral. Key aspects of our ensemble learning approach include using a shared dataset to train all three deep networks, employing a parallel training structure, and utilizing a stacking method where CNN-AEs are trained first, and their outputs are concatenated and fed into the meta-learner (fully connected network). In the training process, 70% of the data were used for training, 15% for testing, and 15% for validation.

**Figure 8:**
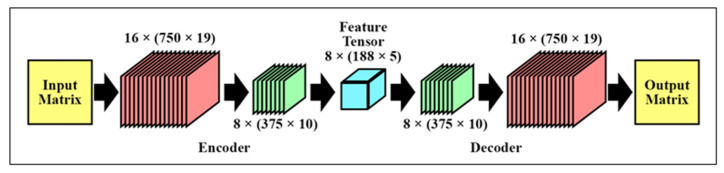
Visualization of the proposed architecture of the stacked autoencoder.

An autoencoder is a type of neural network that uses backpropagation to transform low-dimensional input data into high-dimensional representations by extracting essential information. By minimizing the reconstruction loss between the input and the output, the network creates a compressed representation in its middle layer. The architecture consists of input layers, hidden layers, and output layers, as depicted in Figure 8. The portion of the network from input to hidden layer is termed the “encoder network”, while the segment from hidden layer to output layer is the “decoder network” [82]. Both input and output layers share the same dimensions.

Our proposed encoder network comprises a set of two convolutional blocks that process the MCCSP outputs. Each block contains a convolutional layer with 3 × 3 and 2 × 2 sized kernels, followed by ReLU activation and max-pooling layers. The hidden layer then holds the compressed output, capturing the essential features in a feature tensor. Finally, the decoder network reconstructs the feature tensor using up-sampling and convolutional blocks, which include transpose convolutional layers, ReLU activation, and up-sampling layers. The optimizer used was Adam, and the model was trained over a total of 10 epochs. The transpose convolutional layer operates similarly to a regular convolutional layer but in reverse, effectively increasing the dimensionality of input layers. The autoencoder is trained by minimizing the reconstruction loss function between the original and the reconstructed data. Further details about the autoencoder network are provided in Table 3.

The features extracted from the ensembled autoencoders are reshaped into vectors. Then, these three vectors are concatenated and fed into a fully connected network with 128 neurons. The final output of this network, after passing through the last layer with 3 neurons, is ready for classification. This process is summarized in Table 4.

## 4. Results

In this section, the outcomes of the proposed model will be revealed and contrasted with earlier research. All pertinent simulations were executed on a computer system that includes a Core i7 processor, and 16 GB of RAM (4800 MHz).

**Figure 9:**
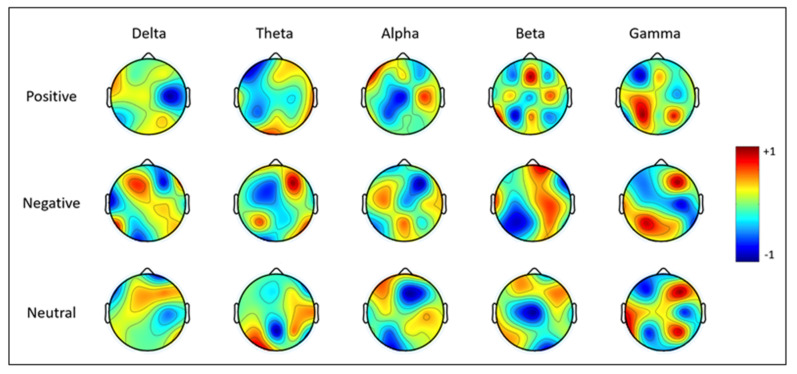
Brain topography after applying MCCSP for different classes and frequency bands.

As mentioned, we used MCCSP as the initial processing. Figure 9 illustrates the topographical distribution of brain signals post-MCCSP processing, categorized by different emotional states and frequency bands. In this figure, each row corresponds to a distinct emotional state (positive, negative, and neutral), while each column represents a specific frequency band (delta, theta, alpha, beta, and gamma). The application of a CSP algorithm within the MCCSP framework has a notable impact on the variance of EEG channels, either amplifying or attenuating the signal variance within specific frequency ranges for each emotional state. For instance, in the alpha band, the frontal region exhibits significant variance in the positive emotional state. Conversely, the frontal and central regions display notable variance in the negative emotional state. In the neutral state, variance is prominently observed in the central region. This variation in signal power, depicted by the color scale from blue (indicating negative variance) to red (indicating positive variance), underscores the spatial modulation of brain activity associated with different emotional states and frequency bands. Note that the signal was normalized between −1 and 1 to be comparable.

**Figure 10:**
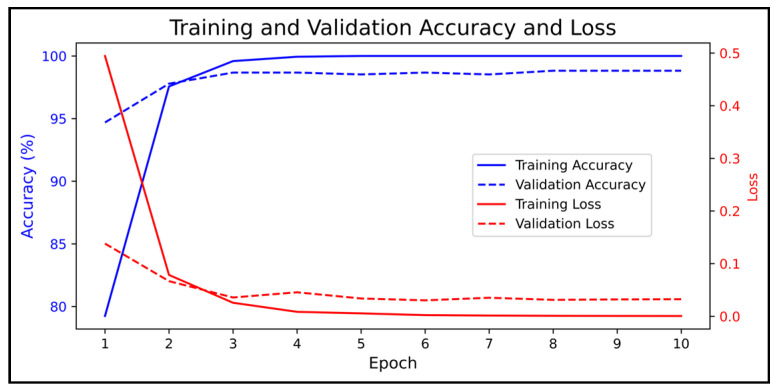
Performance of the proposed model.

Figure 10 illustrates the accuracy and error of the suggested approach for the automatic identification of three emotions, positive, negative, and neutral, for both training and validation sets on 10 iterations of the algorithm. This algorithm is founded on the combination of MCCSP and autoencoder networks. It is widely recognized that the validity of the proposed method was established in four iterations of the algorithm, achieving a 99% accuracy rate. Furthermore, the network error was significantly decreased from 0.5 to 0.001.

**Figure 11:**
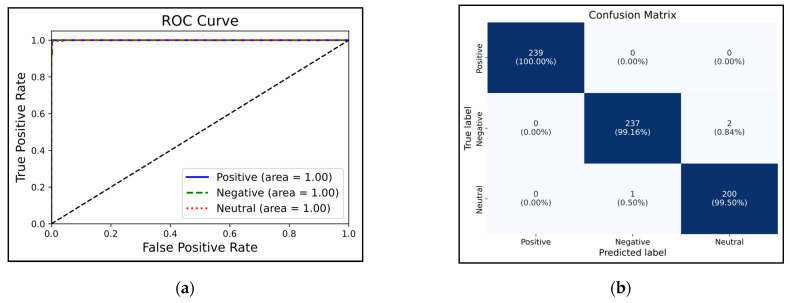
ROC analysis (**a**) and confusion matrix (**b**) based on the proposed model.

**Figure 12:**
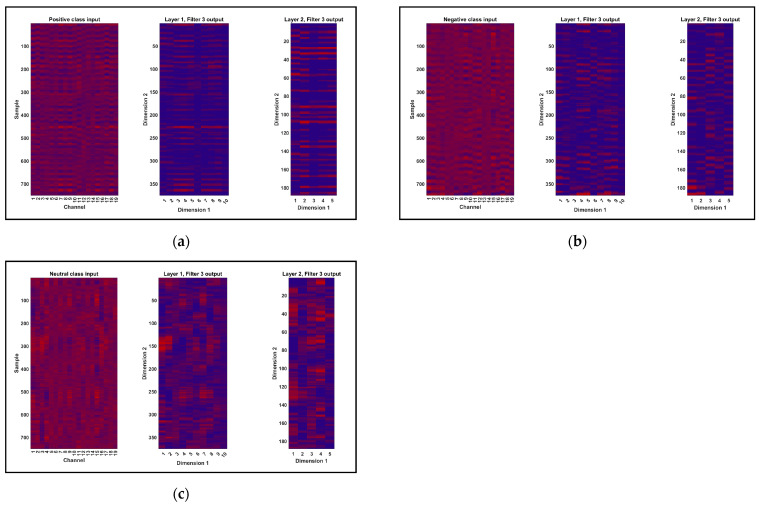
Visualization of a representation of the input data to the network and output of the third filter of the first and second layers for three classes: positive (**a**), negative (**b**), and neutral (**c**).

Figure 11 presents the receiver operating characteristic (ROC) analysis and the confusion matrix for the automatic detection of three emotions. As per this figure (Figure 11a), the optimal placement of the curves for all three emotions lies between 0.9 and 1, signifying the optimal performance of the proposed method’s classification process. Moreover, as per the confusion matrix (Figure 11b), only two instances of negative emotion and one instance of neutral emotion were misclassified, suggesting that the proposed network was highly effective in distinguishing samples of each class. Note that these figures illustrate the all-band situation. Figure 12 is a display of the data of three classes, positive, negative, and neutral, in different layers of the autoencoder network. As can be seen, the input layer data, the output of the third filter from the first CNN layer, and the output of the third filter from the second CNN layer in the autoencoder network for the three emotion classes are shown as examples. By visually comparing the output of the second CNN layer among the classes, the network’s ability to extract distinguishable features is clearly evident.

**Figure 13:**
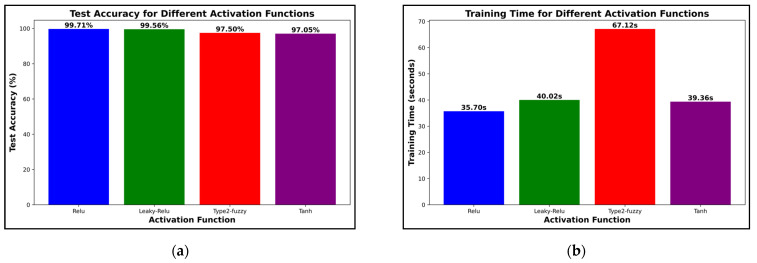
Comparing the accuracy (**a**) and network training time (**b**) with different functions.

To determine the importance of the activation function in the proposed method, three other commonly used functions, namely Leaky-ReLU, Type-2 Fuzzy, and tanh, were examined. The results of this examination are shown in Figure 13. As can be seen, all functions led to an accuracy of over 97%. Among these functions, ReLU and Leaky-ReLU achieved accuracies of 99.71% and 99.56%, respectively, indicating the compatibility of these functions with the proposed method (Figure 13a). However, in examining the time required to train the network, the ReLU function required the least time among the functions (Figure 13b). The ReLU function with 35 s and the Type-2 Fuzzy function, respectively, had the least and most time spent on training the network in the proposed method.

**Figure 14:**
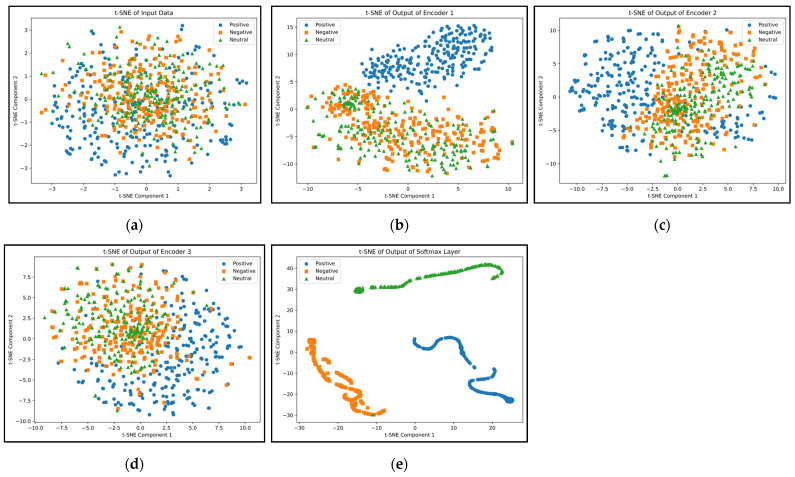
Visual representation of examples for five different layers of the proposed network: input (**a**), output of autoencoders (**b**–**d**), and SoftMax output (**e**).

Figure 14 presents the t-distributed stochastic neighbor embedding (t-SNE) diagram for the three classes of emotions across various network layers. As depicted, nearly all samples from the three classes are entirely distinct from each other in the final layer of the network.

**Figure 15:**
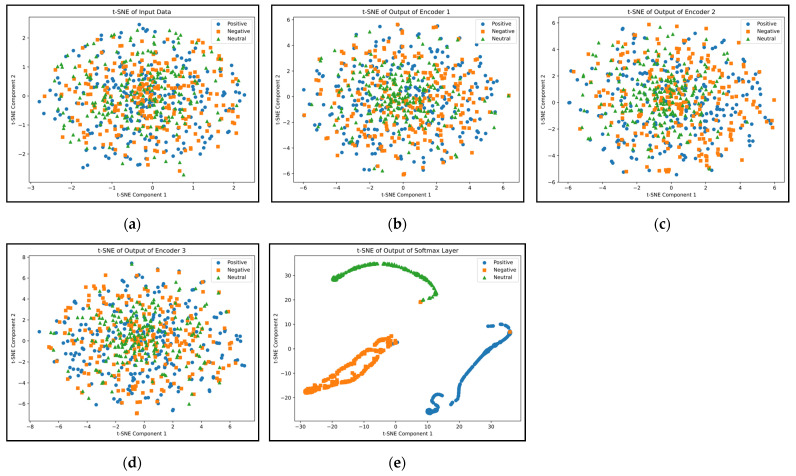
Visual representation of examples for five different layers of the proposed network with −4 dB SNR: input (**a**), output of autoencoders (**b**–**d**), and SoftMax output (**e**).

In addition, the suggested approach underwent testing in a simulated noise-filled environment for additional assessment. Gaussian white noise was incorporated into the collected EEG data at different signal-to-noise ratios (SNRs) for this purpose. The results acquired are illustrated in Table 5. Considering the susceptibility of EEG signals to noise, the network employed for categorizing emotions must be resilient to noise for it to be applicable in real-time scenarios. Figure 15 shows the t-SNE diagram of three emotion classes in different layers of the proposed method at −4 dB SNR. As can be seen, the suggested network can guarantee superior classification precision in environments with noise and is suitable for use in BCI applications.

Also, the performance of the proposed method was tested in different frequency bands to examine the amount of information related to positive, negative, and neutral emotions in these bands. The results of this case are shown in Table 6. As can be seen, the highest classification accuracy is related to the alpha frequency band (99.96%). The speed of training and testing the network is also noteworthy, which for all frequency band divisions is between 36 and 42 s.

## 5. Discussion

As we have seen, deep learning can be applied in various parts of human life, including assessment [83], mental health [84,85], feature extraction [86], and emotion recognition [87]. In this study, we introduced a novel approach to classifying emotions from EEG signals into three categories: positive, negative, and neutral. We collected a custom EEG dataset, specifically designed to capture emotional responses induced by musical stimuli. Utilizing the MCCSP technique for initial signal processing, we enhanced feature extraction by identifying spatial patterns across different classes. This method allowed us to capture the intricate spatial–temporal dynamics of EEG signals, which are crucial for accurate emotion recognition. Our ensemble model, comprising three autoencoders with CNN layers, demonstrated significant improvements over traditional methods. The parallel structure and stacking method employed in our framework enabled the model to effectively handle the complexity and high dimensionality of EEG data. This approach not only reduced the need for manual feature engineering but also enhanced the robustness and accuracy of emotion classification. Our proposed method achieved an impressive classification accuracy of 99.5%, outperforming previous studies and demonstrating its potential for real-time BCI applications. The key strengths of our method include its ability to automatically extract and process complex features, resilience to noise, and superior performance in classifying emotions across various frequency bands. These findings align with recent developments in CSP-based techniques, which have proven useful in multiple BCI applications [88,89,90].

Some studies have shown that higher frequency spectrums such as beta and gamma are more effective in identifying emotions [91,92]. Therefore, we divide the beta and gamma frequency bands into sub-bands beta1 (13–21 Hz), beta2 (21–30 Hz), gamma1 (30–38 Hz), and gamma2 (38–45 Hz) to further investigate the performance of the proposed method in these spectrums. Table 7 shows the result of this investigation. As can be seen, the gamma2 frequency sub-band shows the highest accuracy for classifying three emotions (97.94%), which is comparable to the accuracy of other bands (Table 6). However, the beta sub-bands resulted in lower accuracy, which is still noteworthy for a three-class problem. Although both beta and gamma bands play a role in experiencing emotions and processing information, the gamma band is more specifically associated with complex information processing and sensory coherence, which can include deep emotional experiences [93]. This might explain the lower accuracy associated with the beta band.

Table 8 contrasts previous studies and their respective methods with the proposed model. According to Table 8, the proposed method achieved the highest accuracy compared to earlier works. However, this comparison may not be entirely fair as the databases used are not the same. 

**Figure 16:**
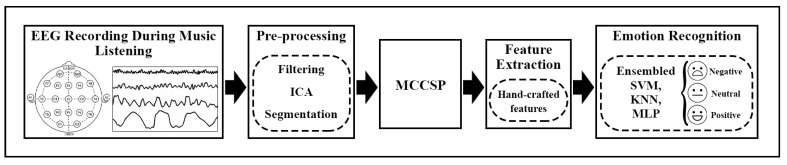
Traditional ensemble method framework.

To fairly compare the performance of the proposed method with other methods, it is essential to apply all of them to the same dataset. For this purpose, we developed another approach, referred to as the traditional method, for the sake of comparison with our proposed method. The framework and extracted features of this method are depicted in Figure 16 and Table 9, respectively. The features, after extraction, were tested for their significance using the Kruskal–Wallis statistical test with a confidence level of 0.05. For the classification section, three prevalent classifiers, SVM, KNN, and MLP, were employed in a bootstrap ensemble model to yield the highest accuracy. The kernel of the SVM was linear. The number of neighbors for the KNN and the number of hidden layers for the MLP network were set to 5 and 35, respectively. To delve deeper, the accuracy of the traditional ensemble method was evaluated across various frequency bands and documented in Table 10. As per the table, the highest accuracy is associated with the beta frequency band (95.90%). By juxtaposing Table 6 and Table 10, we deduce that our suggested method outperforms the traditional method across different frequency bands, particularly all-bands (approximately 5% more).

Table 11 assesses the proposed model against previous studies based on this dataset to gauge its effectiveness. The proposed emotion-recognition method achieved a classification accuracy of approximately 99.44 ± 0.39%, while studies [13,28] reported accuracies of around 95.23% and 98%, respectively.

## 6. Conclusions

In this study, we present a new framework for the automatic detection of emotions from EEG signals by integrating MCCSP and an ensemble network consisting of three parallel autoencoders with CNN layers and ReLU activation functions. For this purpose, we collected a standard dataset, comprising EEG signals of individuals listening to music. The proposed model demonstrated a remarkable accuracy of 99.44 ± 0.39% in distinguishing between positive, negative, and neutral emotional classes. The high level of accuracy is particularly important for applications in neuromarketing and user experience design, where accurate emotion detection is crucial. In these fields, understanding and responding to users’ emotional states with precision can greatly enhance product engagement and customer satisfaction [96,97].

In addition, the proposed method exhibited significant robustness in a simulated noisy environment, maintaining accuracy above 97% even at an SNR of −4 dB. This robustness is essential for real-world applications, such as adaptive learning systems, where environmental noise can be a common challenge [98]. Adaptive learning systems personalize the learning experience based on the learner’s performance, behavior, and emotional state. Accurate emotion recognition helps these systems adapt content and teaching methods to keep students engaged and motivated.

One of the notable strengths of our model is its rapid processing speed for test data, with an average detection time of approximately 0.35 s. This swift response is crucial for applications in mental health monitoring, where timely detection of emotional states can significantly impact the effectiveness of interventions. Mental health monitoring systems can benefit from such rapid emotion recognition by providing real-time feedback and support to individuals. For instance, detecting signs of stress, anxiety, or depression early allows for immediate intervention, thereby improving mental health outcomes. The ability to swiftly and accurately detect emotional states ensures that these systems can respond to changes in a person’s emotional well-being promptly, making them more effective and reliable in practical scenarios [99,100].

Despite our study’s promising results, there are some limitations. Firstly, the number of subjects in our dataset was relatively small, which might affect the generalizability of our findings. In order to address this, future research should aim to collect larger and more diverse datasets. Expanding the dataset not only increases the statistical power of the study but also enhances the robustness of the model across different populations and conditions.

Secondly, our analyses were not gender-based, which could overlook potential gender differences in emotional responses. To overcome this limitation, future studies should include a balanced representation of genders and perform gender-specific analyses. This approach would help in understanding any gender-related variations in emotion recognition and improving the model’s accuracy and applicability for all genders.

Thirdly, the selection of musical stimuli was limited to Iranian songs due to cultural preferences and availability. This restriction may affect the generalizability of our findings to other cultural contexts. To address this limitation, future research should consider including a diverse range of musical stimuli from different cultures. This approach would help in understanding how different types of music influence emotional responses and provide evidence that the model can generalize well across various cultural backgrounds. Additionally, collaborating with researchers from different cultural contexts can provide a richer and more diverse dataset, enhancing the model’s robustness and applicability.

## Figures and Tables

**Table 1:** Validation of subjects in the EEG signal recording process for emotions recognition.

Subject	Sex	Age	BDI	Mean Valence of Induction for Positive Emotion	Mean Arousal of Induction for Positive Emotion	Mean Valence of Induction for Negative Emotion	Mean Arousal of Induction for Negative Emotion	Result of Validation	Reason for Removal Subject
1	M	25	16	9	9	1.8	1	✔	-
2	M	24	22	6.8	6.2	3.6	2	✘	Beck depression (21 < 22)
3	F	27	19	6.2	7.4	4.2	4.6	✘	Mismatch of the control question in the SAM test
4	M	24	4	7.4	7.6	2.4	2.6	✔	-
5	M	24	0	5.8	5	4.4	5.6	✘	Mismatch of the control question in the SAM test
6	M	28	10	5.6	5.4	2	1.6	✘	The desired lack of induction in the positive emotional class
7	M	28	13	7.2	7.4	3.8	3.8	✘	The desired lack of induction in the negative emotional class
8	M	20	19	7.8	7.4	2.8	3	✔	-
9	M	26	9	7.4	7	3.4	5.4	✘	The desired lack of induction in the negative emotional class
10	F	23	9	6.8	6.6	3.8	3.2	✘	The desired lack of induction in the negative emotional class
11	F	25	22	7.8	8	4.5	3	✘	Beck depression (21 < 22)
12	F	27	1	8.6	8.6	2	1.2	✔	-
13	F	29	9	6	6	2	1.2	✔	-
14	M	26	8	8	8	1.8	1.8	✔	-
15	F	25	12	-	-	-	-	✘	Motion noise
16	M	27	0	7.4	8	1.8	2	✔	-

**Table 2:** The sequence and musical genres employed for eliciting emotional responses.

Emotion Sign and Music Number	The Type of Emotion Created in the Subject	The Style of the Music
N1	Negative	Advance income of Isfahan
P1	Positive	Azari 6/8
N2	Negative	Advance income of Homayoun
P2	Positive	Azari 6/8
P3	Positive	Bandari 6/8
N3	Negative	Afshari piece
N4	Negative	Advance income of Isfahan
P4	Positive	Persian 6/8
N5	Negative	Advance income of Dashti
P5	Positive	Bandari 6/8

**Table 3:** The details of the autoencoder network architecture.

L	Layer Type	Activation Function	Output Shape	Size of Kernel and Pooling	Number of Filters	Padding (Size: Same)
0–1	Convolution 2-D	ReLU	(750, 19, 16)	3 × 3	16	Yes
1–2	Max-pooling 2-D	-	(375, 10, 16)	2 × 2	-	Yes
2–3	Convolution 2-D	ReLU	(375, 10, 8)	2 × 2	8	Yes
3–4	Max-pooling 2-D	-	(188, 5, 8) ^1^	2 × 2	-	Yes
4–5	T. Convolution 2-D ^2^	RuLU	(188, 5, 8)	2 × 2	8	Yes
5–6	Up-sampling 2-D	-	(375, 10, 8)	2 × 2	-	Yes
6–7	T. Convolution 2-D	ReLU	(375, 10, 16)	3 × 3	16	Yes
7–8	Up-sampling 2-D	-	(750, 19, 16)	2 × 2	-	Yes
8–9	T. Convolution 2-D	Sigmoid	(750, 19)	3 × 3	1	Yes

^1^ Feature tensor dimension, ^2^ Transpose Convolution 2-D.

**Table 4:** The details of the output of ensemble model.

Layer Type	Activation Function	Output Shape
Flatten	-	(7520, 1)
Concatenate	-	(22,560, 1)
Fully connected	ReLU	(128, 1)
Fully connected	SoftMax	(3, 1)

**Table 5:** The accuracy of the proposed method at different SNRs.

SNR (dB)	Accuracy (%)
−4	97.73
0	97.91
1	97.94
10	98.54
20	98.82

**Table 6:** The performance of the proposed method in different frequency bands.

Freq. Band	Acc.	Pre.	Rec.	F1	Kappa	Time (s)	
						Train	Test
Delta	98.97	99.03	99.02	99.01	98.45	35.89	0.35
Theta	98.97	98.92	98.94	98.93	98.45	39.94	0.34
Alpha	99.96	99.96	99.96	99.96	99.93	36.22	0.33
Beta	99.52	99.49	99.51	99.49	99.27	35.86	0.32
Gamma	99.64	99.59	99.64	99.62	99.45	35.38	0.35
All-bands	99.56	99.53	99.56	99.54	99.33	40.98	0.35

**Table 7:** The performance of the proposed method in beta, and gamma frequency sub-bands.

Freq. Band	Acc.	Pre.	Rec.	F1	Kappa
Beta1	75.70	76.09	75.30	75.48	63.59
Beta2	77.47	78.35	78.61	78.48	66.11
Gamma1	92.78	92.62	92.72	92.65	89.15
Gamma2	97.94	97.92	98.05	97.98	96.90

**Table 8:** Comparing the performance of prior research with the proposed model.

Study	Stimulus	Ensemble Method	Number of Emotions Considered	Acc. (%)
Bhatti et al. [94]	Music	WT + MLP	4	78.11
Subasi et al. [95]	Video clip	RFE + SVM	3	93
Salama et al. [75]	Video clip	3D-CNN	2	96.46
Ashokkumar et al. [76]	Video clip	RNN	2	94.67
Iyer et al. [77]	Video clip	CNN + LSTM + Hybrid	3	97.16
Bagherzadeh et al. [80]	Video clip	AlexNet + VGG-19 + Inception-v1 + ResNet-18 + Inception-v3	4	96.90
Proposed method	Music	MCCSP + CNN-AE	3	99.5

**Table 9:** Extracted features in traditional method.

Features Extracted from MCCSP Output	Number
Variance of the samples for each channel	19
Mean autocorrelation across channels	1
Mean entropy of autocorrelation across channels	1
Autocorrelation zero-crossings across channels	1
Statistical features * of DWT	10
Total	32

* The mean, standard deviation, median, maximum, minimum, absolute sum, and the moments of the second (variance), third (skewness), fourth (kurtosis), and fifth orders.

**Table 10:** The performance of the traditional ensemble method in different frequency bands.

Freq. Band	Acc.	Pre.	Rec.	F1	Kappa
Delta	94.58	94.53	94.61	94.55	91.87
Theta	93.47	93.53	93.43	93.46	90.22
Alpha	93.58	93.81	93.61	93.55	90.39
**Beta**	**95.90**	**95.89**	**95.84**	**95.85**	**93.86**
Gamma	94.24	94.59	94.59	94.48	91.38
All-bands	94.14	93.86	93.79	93.80	91.22

**Table 11:** Performance comparison of prior research and the proposed model on the same dataset.

Study	Method	Number of Emotions Considered	Acc. (%)
Sheykhivand et al. [28]	CNN-LSTM	3	95.23
Baradaran et al. [13]	Customized CNN	3	98
Baradaran et al. [96]	CNN + Type-2 Fuzzy	2	98.2
Traditional method	MCCSP + Hand-Crafted Features	3	94.14
**Proposed method**	**MCCSP + CNN-AE**	**3**	**99.44 ± 0.39**

## Data Availability

The raw data supporting the conclusions of this article will be made available by the authors on request.
